# Relationship between Disease Characteristics and Circulating Interleukin 6 in a Well-Characterized Cohort of Patients with Systemic Lupus Erythematosus

**DOI:** 10.3390/ijms241814006

**Published:** 2023-09-12

**Authors:** Julia Mercader-Salvans, María García-González, Fuensanta Gómez-Bernal, Juan C. Quevedo-Abeledo, Antonia de Vera-González, Alejandra González-Delgado, Raquel López-Mejías, Candelaria Martín-González, Miguel Á. González-Gay, Iván Ferraz-Amaro

**Affiliations:** 1 Division of Dermatology, Hospital Universitario de Canarias, 38320 Tenerife, Spain; juliamercader96@gmail.com; 2 Division of Rheumatology, Hospital Universitario de Canarias, 38320 Tenerife, Spain; margagon23@hotmail.com; 3 Division of Central Laboratory, Hospital Universitario de Canarias, 38320 Tenerife, Spain; fuensanta95@gmail.com (F.G.-B.); adeverag@gmail.com (A.d.V.-G.); alejandra.gd88@gmail.com (A.G.-D.); 4 Division of Rheumatology, Hospital Doctor Negrín, 35010 Las Palmas de Gran Canaria, Spain; quevedojcarlos@yahoo.es; 5 Epidemiology, Genetics and Atherosclerosis Research Group on Systemic Inflammatory Diseases, IDIVAL, 39011 Santander, Spain; rlopezmejias78@gmail.com; 6 Division of Internal Medicine, Hospital Universitario de Canarias, 38320 Tenerife, Spain; mmartgon@ull.edu.es; 7 Department of Internal Medicine, University of La Laguna (ULL), 38200 Tenerife, Spain; 8 Division of Rheumatology, IIS-Fundación Jiménez Díaz, 28040 Madrid, Spain; 9 Department of Medicine, University of Cantabria, 39005 Santander, Spain

**Keywords:** interleukin-6, systemic lupus erythematosus, disease damage, complement system, SCORE2

## Abstract

Interleukin-6 (IL-6) is a proinflammatory cytokine that mediates pleiotropic functions in immune responses and inflammatory diseases. The literature lacks studies, with a clinical perspective, on the relationship between IL-6 serum levels and the characteristics of the disease in patients with systemic lupus erythematosus (SLE). In the present work, we aimed to analyze the association between circulating IL-6 and disease manifestations in a well-characterized series of patients with SLE. Serum IL-6 levels and disease activity (SLEDAI-2K), severity (Katz) and damage index (SLICC-DI), complete lipid profile, and subclinical carotid atherosclerosis were evaluated in 284 patients with SLE. In addition, a complete characterization of the complement system was performed in samples from patients with SLE. A multivariate linear regression analysis was carried out to study the relationship between clinical and laboratory characteristics of the disease and IL-6 levels. Age (beta coef. 0.07 [95%CI 0.01–0.1] pg/mL, *p* = 0.014), C-reactive protein (beta coef. 0.21 [95%CI 0.16–0.25] pg/mL, *p* < 0.01), and male gender (beta coef. 2 [95%CI 0.3–0.5] pg/mL, *p* = 0.024), were positively associated with higher IL-6 levels in SLE patients. Most disease characteristics and damage and activity indices did not show significant relationships with IL-6. However, after multivariate analysis, IL-6 was associated with lower serum levels of HDL cholesterol (beta coef. −0.04 [95%CI −0.08–(−0.1)] pg/mL, *p* = 0.011), and apolipoprotein A1 (beta coef. −0.02 [95%CI −0.04–(−0.001)] pg/mL, *p* = 0.035). In contrast, the alternative complement cascade, C1inh, and C3a were all positively and independently associated with higher serum levels of IL-6. Moreover, stratification of the Systematic Coronary Risk Assessment 2 (SCORE2) results according to different categories of cardiovascular risk was associated with higher circulating serum IL-6 levels (beta coef. 0.2 [95%CI 0.02–0.4], pg/mL, *p* = 0.028). In conclusion, in a large series of SLE patients, IL-6 was not associated with disease-related features of SLE, including damage, severity, or activity indices. However, an association was found between serum IL-6 levels and circulating C3a and cardiovascular risk. Our study emphasizes the importance that IL-6 could have in cardiovascular disease and complement system disruption of SLE patients. Therapies targeting IL-6 could have a role in these two clinical manifestations of patients with SLE.

## 1. Introduction

Systemic lupus erythematosus (SLE) is a chronic autoimmune disease characterized by various manifestations, frequent flare-ups, and the involvement of multiple organs. Patients display a wide range of clinical symptoms, ranging from mild joint and skin problems to severe complications involving the kidneys, blood, and central nervous system [1,2]. The etiology of SLE remains unknown but is clearly multifactorial, with many observations suggesting a role for genetic [3], hormonal [4], immunologic [5,6], and environmental factors [7]. Furthermore, SLE is primarily a disease with abnormalities in immune regulation, including the formation of autoantibodies and immune complexes [8]. Phagocytosis and clearance of immune complexes, apoptotic cells, and necrotic cell-derived material are defective in SLE, allowing the persistence of antigens and immune complexes. This leads to abnormal cell persistence, autophagy, and cytokine production [9]. Furthermore, accelerated atherosclerosis and increased risk of cardiovascular disease have been identified as major causes of morbidity and mortality in patients with SLE [10].

Interleukin-6 (IL-6) is a proinflammatory cytokine that plays a crucial role in various immunological processes associated with host infection, inflammatory disorders, hematopoiesis, and oncogenesis. IL-6 functions as a regulator of the immune response, influencing the proliferation and differentiation of T cells, as well as the final maturation of B cells [11]. Additionally, IL-6 activates macrophages and osteoclasts and is considered a key stimulator of acute phase reactants [12]. Working in conjunction with tumor necrosis factor-alpha and IL-1, IL-6 promotes the production of vascular endothelial growth factor and metalloproteinases. In addition, IL-6, together with transforming growth factor beta, also plays a key role in the generation of subsets of peripherally induced CD4+ and CD8+ cytokine-producing suppressor cells [13]. Furthermore, the role of IL-6 in autoimmunity has been suggested [14]. This has been attributed to the idea that the immune system tightly regulates Th17/Treg cell homeostasis through the IL-6 axis, and the disturbance of this balance causes autoimmunity [15]. However, the role of IL-6 in the pathogenesis of SLE is not fully understood.

The literature lacks studies with a large number of SLE patients in which the relationship between a complete characterization of disease features and serum levels of IL-6 has been studied. In the present work, we studied a considerable and well-characterized series of patients with SLE. In addition to the comprehensive evaluation of clinical and laboratory features, including lipid profile and assessment of insulin resistance, we also analyzed all three complement pathways. In addition, we estimated the cardiovascular risk using the Systematic Coronary Risk Assessment 2 (SCORE2) algorithm and carotid ultrasound to determine subclinical atherosclerosis. After this, we set out to study the relationship between all these characteristics and the serum levels of IL-6. If the expression of IL-6 was found to be associated with specific disease features, then the potential use of therapies targeting this interleukin in SLE could be suggested.

## 2. Results

### 2.1. Demographics and Disease-Related Data on Systemic Lupus Erythematosus Patients

The median (IQR) serum level of IL-6 in SLE patients was 3.5 (IQR 2.3–5.4) pg/mL. Table 1 provides an overview of the characteristics of the 284 patients included in this study. Most of the participants were women (92%), with a mean age ± SD of 50 ± 12 years. The average body mass index was 28 ± 6 kg/m^2^, and the abdominal circumference was 92 ± 14 cm. Classic cardiovascular risk factors included current smoking in 24% of patients, hypertension in 39%, and obesity in 30%. Additionally, 25% of the patients were taking statins and 29% were taking aspirin (Table 1).

The median disease duration was 16 (IQR 7–24) years. The majority of SLE patients had no activity (40%) or mild to moderate activity (39%), as indicated by the SLEDAI-2K score. The SLICC-SDI and Katz indices were 1 (IQR 0–2) and 2 (IQR 1–4), respectively. A SLICC-SDI score of 1 or higher was found in 68% of the patients. Half of the patients (50%) were taking prednisone, with a median daily dose of 5 mg/day (IQR 5–7.5). At the time of recruitment, 67% of patients tested positive for anti-DNA antibodies, and 69% tested positive for anti-ENA antibodies, where anti-SSA was the most detected antibody (35%). Hydroxychloroquine was being used by 69% of the patients at the time of this study. Other less frequently used disease-modifying antirheumatic drugs included methotrexate (11%) and azathioprine (15%). Additional data on SLE-related information can be found in Table 1.

### 2.2. Demographic and Disease Characteristics in Relation to Serum IL-6 Levels

In the univariate analysis, age, waist-hip ratio, and serum CRP levels had a positive and significant relationship with IL-6. Furthermore, the female gender was associated with significantly lower circulating levels of IL-6 compared with male patients (beta coef. −2 [95%CI −5–(−0.03)] pg/mL, *p* = 0.024) (Table 2). Regarding disease-related data, the presence of lupus anticoagulant was the only disease feature that showed a significant association with IL-6 (beta coef. 2 [95%CI 0.3–3] pg/mL, *p* = 0.016). In contrast, the autoantibody profile, the use of various therapies, and the SLICC-DI, SLEDAI-2K, and Katz indices did not reveal any association with serum IL-6 levels.

Since the activity score and the damage and disease severity indices are a sum of different aspects of SLE, we analyzed the relationship between each item of these scores and IL-6 (Table 3). Regarding the Katz index, no associations were found between the items of this score and IL-6.

Similarly, SLEDAI-2K score items were generally not related to circulating IL-6. In this regard, only the presence of pyuria, which was present in 11 patients (4%), was the item that showed a significant relationship with higher levels of circulating IL-6. With respect to SLICC-SDI areas, only the cataract (n = 29, 11%), pleural fibrosis (n = 1, 0%), and skin ulceration (n = 4, 1%) items were significantly related to higher values of serum IL-6 (Table 3 and Table A1).

### 2.3. Multivariate Analysis of the Relationship between Cardiovascular-Related Factors and IL-6

Values for lipid-related molecules, insulin resistance indices, cIMT, the presence of carotid plaque, and the SCORE2 in SLE patients are shown in Table 4.

Regarding lipid profile molecules, after the multivariate analysis, serum levels of total and HDL cholesterol (beta coef. −0.04 [95%CI −0.08–(−0.1)] pg/mL, *p* = 0.011) and apolipoprotein A1 (beta coef. −0.02 [95%CI −0.04–(−0.001)] pg/mL, *p* = 0.035) were significantly associated with lower IL-6 values. The presence of carotid plaque and cIMT and the insulin resistance indices were unrelated to IL-6 after multivariate adjustment. However, SCORE2 results, considered as a continuous variable, were associated with significantly higher serum IL-6 levels (beta coef. 0.2 [95%CI 0.02–0.4], pg/mL, *p* = 0.028) (Table 4).

### 2.4. Multivariate Analysis of the Relationship between the Pathways and Components of the Complement System and IL-6 

A full characterization of the complement system using an assessment of serum values for C1q, C2, C3, C3a, factors H and D, and C1-inhibitor, as well as functional assays of the three classical, alternative, and lectin pathways is listed in Table 5. After multivariate analysis, the alternative complement cascade and C1inh and C3a were found to be positively and independently associated with higher serum levels of IL-6 (Table 5).

## 3. Discussion

To date, our work represents the most comprehensive characterization of IL-6 serum levels in patients with SLE. According to our findings, IL-6 levels do not show a relationship with most of the characteristics directly related to the disease when this association is studied cross-sectionally. However, other characteristics such as the lipid profile and the SCORE2 cardiovascular risk algorithm did show a relationship with IL-6. Of note, the alternative pathway of the complement system and C3a were also positively related to IL-6.

A recent meta-analysis reviewed the relationship between serum IL-6 levels and disease activity in SLE [16], which included 17 previous studies on this topic. According to the meta-analysis, IL-6 levels were significantly higher in SLE than in healthy controls. Furthermore, it showed a significant positive correlation between IL-6 levels and disease activity scores. In contrast, in our largest fully characterized series, we did not observe a relationship between various disease scores, including activity, damage, or severity, and IL-6.

A possible explanation for our findings could tight disease control since most of our patients were in the mild or no disease activity categories when SLEDAI-2k was applied. The literature is scarce regarding the relationship between IL-6 and specific manifestations of the disease. In this sense, urinary IL-6 levels in 29 patients with active class IV lupus nephritis were significantly higher than in patients with other classes of lupus nephritis [17]. After treatment, urinary levels of IL-6 decreased significantly. Furthermore, IL-6 was shown to be increased in the cerebrospinal fluid of patients with lupus psychosis [18,19] and to have an inverse correlation with hemoglobin levels in 171 patients with SLE [20]. However, the number of patients recruited in these studies was less than in our series, and none of the studies performed multivariate adjustments.

Remarkably, IL-6 and CRP highly correlated in our study. Hepatocytes are responsible for producing significant amounts of CRP, primarily triggered by IL-6 [21]. Due to this, this association is expected to occur. However, it is believed that autoimmune diseases where the type I interferon gene signature predominates, such as SLE, deviate from the usual pattern where CRP levels typically correlate with the degree of inflammation [22]. Two old previous reports, with a small number of patients, described the lack of association of CRP and IL-6 in patients with SLE [23,24]. This was not the case of our study. Moreover, CRP has been linked with cardiovascular disease in general population [25]. For this reason, the fact that there is a relationship between IL-6 and cardiovascular disease in our work could be mediated by the interrelation between both IL-6 and CRP.

Emerging research has provided substantial evidence indicating that IL-6 expression exhibits varying degrees of elevation in cardio-cerebrovascular conditions, such as atherosclerosis, myocardial infarction, heart failure, and ischemic stroke [26]. This cytokine actively contributes to the onset and progression of cardiovascular disease, particularly in response to triggers like ischemia, hypoxia, oxidative stress, inflammation, and vascular occlusion. This was supported by two large meta-analyses that confirmed the crucial role played by a variant allele of the *IL6R* gene encoding the IL-6 receptor in the generation of inflammation and the associated risk of coronary heart disease [27,28]. Furthermore, the hypothesis that IL6 is a potential target in cardiovascular disease is being currently tested in the ZEUS trial, which is being conducted to determine if ziltivekimab (a human anti-IL-6 monoclonal antibody) reduces the risk of cardiovascular events in people with cardiovascular disease, chronic kidney disease, and inflammation. In our study, we found a positive relationship between the SCORE2 cardiovascular risk calculation algorithm and IL-6. In addition, after adjusting for covariates, IL-6 was associated with lower levels of HDL cholesterol and apolipoprotein A1. Therefore, we believe that in patients with SLE, IL-6 may retain the deleterious role in cardiovascular disease that has been shown in the general population.

Our study is the first in the literature to evaluate the relationship between a complete characterization of the complement system and serum IL-6 levels. In this regard, after multivariate adjustment, we observed a relationship between IL-6 and the complement particles C3a and C1inh and the alternative complement pathway. Cytokines, such as IL-6, are typically released in sites of inflammation and then travel via circulation to the liver, where they increase the hepatic synthesis of complement proteins. C1inh is a known regulator of the complement system. It prevents excessive complement activation on a target as well as in plasma. This function is performed by binding to each C1r and C1s subcomponent of the C1 complex. Furthermore, the complement system promotes the inflammatory response primarily through the liberation of C3a anaphylatoxin. The positive association described in our work between IL-6 and these two complement elements matches with the pro-inflammatory role of IL-6 and its role in the stimulation of complement protein synthesis. The relationship with the alternative cascade, and not with the classic one, is notable. The alternative pathway is dominant over the classical pathway and the lectin pathway under normal physiological conditions. The continued activation of C3 by the alternative pathway is called “tick over”, which generates low levels of C3a and is amplified if necessary [29]. It is possible that IL-6 could be more related to this production of complement and not to the activation/consumption that occurs in the disease.

## 4. Limitations

We acknowledge that we did not include healthy controls in our study. However, the comparison with controls was studied in previous work [13]. In addition, the design of our study was cross-sectional and, therefore, causality cannot be inferred from our results. Furthermore, because SLE is characterized by complexities in disease phases, flares, pathological states, treatment effects, etc., the cross-sectional design of our work did not allow us to identify how IL-6 varies during the disease progression.

## 5. Conclusions

In conclusion, IL-6 is not related to the damage, activity, or severity produced by SLE. However, certain abnormalities in complement system cascades are associated with IL-6. Furthermore, a positive relationship between cardiovascular risk and IL-6 exists. These findings are relevant since they could support the use of anti-IL-6 therapies for certain manifestations of the disease, such as complement system disruption, as well as the cardiovascular disease that frequently accompanies SLE.

## 6. Materials and Methods

### 6.1. Study Participants

Two hundred and eighty-four patients with SLE were assessed in a cross-sectional study. All patients were 18 years or older and met ≥ 4 American College of Rheumatology (ACR) classification criteria for SLE [30]. A diagnosis of SLE was performed by rheumatologists, and all the patients were regularly followed up in rheumatology outpatient clinics. Patients taking a prednisone dose equal to or lower than 10 mg/day were allowed to participate in this study since glucocorticoids are often used in the management of SLE. The research was conducted in accordance with the Declaration of Helsinki. The study protocol was approved by the Institutional Review Committee at Hospital Universitario de Canarias and at Hospital Universitario Doctor Negrín (both in Spain), and all patients provided informed written consent (Approval Number 2015_84).

### 6.2. Data Collection

The patients included in this study completed a questionnaire on cardiovascular risk factors and medication use and underwent a physical examination. Weight, height, body mass index, abdominal circumference, and systolic and diastolic blood pressure (measured with the participant in a supine position) were assessed under standardized conditions. Information regarding smoking status and hypertension treatment was obtained from the questionnaire. Medical records were reviewed to verify specific diagnoses and medications. SLE disease activity and damage were assessed using the Systemic Lupus Erythematosus Disease Activity Index−2000 (SLEDAI-2K) [31] and the Systemic Lupus International Collaborating Clinics/American College of Rheumatology (SLICC/ACR Damage Index -SDI-) [32], respectively. For the present study proposal, the SLEDAI-2k index was divided into none (0 points), mild (1–5 points), moderate (6–10 points), high (11–19), and very high activity (>20), as previously described [33]. The severity of the disease was measured using the Katz index [34]. In addition, carotid ultrasonography was performed to assess carotid intima-media wall thickness (cIMT) in the common carotid artery and to identify focal plaques in the extracranial carotid according to Mannheim consensus definitions [35]. In this study, HOMA2, the updated computer HOMA model, was utilized [36]. Also, the SCORE2 risk prediction algorithm, an updated model to estimate the 10-year risk of cardiovascular disease in Europe, was assessed in this cohort of patients [37]. Human IL-6 was measured using the electrochemiluminescence immunoassay method (Roche Diagnostics, Indianapolis, IN, USA).

### 6.3. Complement System Assessment

The SVAR functional complement assays under the Wieslab^®^ brand (Sweden) were used to assess classical, alternative, and lectin pathways activity. These tests combine principles of the hemolytic assay for complement function with the use of labeled antibodies specific for the neoantigen produced as the result of C activation. The amount of neoantigen generated is proportional to the functional activity of C pathways. Microtiter strip wells are coated with classical, alternative, or lectin pathway-specific activators. The patient’s serum is diluted in a diluent containing a specific blocker to ensure that only the studied pathway is activated. The specific coating activates C during the incubation of the diluted patient serum in the wells. The wells are then washed, and C5b-9 is detected with an alkaline phosphatase-labeled specific antibody against the neoantigen expressed during membrane attack complex (MAC) formation. After an additional washing step, the detection of specific antibodies is obtained using incubation with an alkaline phosphatase substrate solution. The amount of C activation correlates with the intensity of the color and is measured in terms of absorbance (optical density). The amount of formed MAC (neo-epitope) reflects the activity of the C cascade. The result is expressed semi-quantitatively using the optical density ratio between a positive control and the sample. The classical, alternative, and lectin cascade values should be interpreted as the lower the level, the greater the activation of the pathway. Wieslab^®^ has validated these functional assays by studying their correlation and concordance with the classical CH50 and AH50 hemolytic tests (https://www.svarlifescience.com/ accessed on 15 April 2023). C2, C3, C3a, C4, and C1q were analyzed using turbidimetry (Roche), the C1-inhibitor was analyzed using nephelometry (Siemens), and factor D and factor H were assessed using an enzyme-linked immunosorbent assay (ELISA Duoset, R&D). Both the intra- and inter-coefficients of variability were <10% for these assays.

### 6.4. Statistical Analysis

Demographic and clinical characteristics in patients with SLE were described as mean ± standard deviation (SD) or percentages for categorical variables. For non-normally distributed continuous variables, data were expressed as the median and interquartile range (IQR). The relationship between disease characteristics and circulating IL-6 was evaluated using multivariate linear regression analysis. Univariate relations with a *p*-value less than 0.20 were adjusted for covariates. In the analysis of the association between the complement system, cardiovascular risk factors, and IL-6, confounders were selected from demographics if they had a relationship with both the independent and dependent variables and a *p*-value less than 0.20. All the analyses used a 5% two-sided significance level and were performed using Stata software, version 17/SE (StataCorp, College Station, TX, USA). *p*-values < 0.05 were considered statistically significant.

## Figures and Tables

**Table 1:** Characteristics of patients with SLE included in this study.

	SLE Patients
	(n = 284)
Interleukin-6, pg/mL	3.5 (2.3–5.4)
Age, years	50 ± 12
Female, n (%)	261 (92)
Body mass index, kg/m^2^	28 ± 6
Abdominal circumference, cm	93 ± 14
Hip circumference, cm	103 ± 12
Waist-to-hip ratio	0.90 ± 0.07
Systolic pressure, mmHg	127 ± 20
Diastolic pressure, mmHg	79 ± 11
Cardiovascular co-morbidity	
Smoking, n (%)	69 (24)
Diabetes, n (%)	18 (6)
Hypertension, n (%)	111 (39)
Obesity, n (%)	85 (30)
Statins, n (%)	72 (25)
Aspirin, n (%)	80 (29)
SLE related data	
Disease duration, years	16 (7–24)
CRP, mg/dl	2.0 (0.8–4.4)
SLICC-DI	1 (0–2)
SLICC-DI ≥ 1, n (%)	191 (68)
Katz Index	2 (1–4)
Katz ≥ 3, n (%)	126 (44)
SLEDAI-2K	2 (0–4)
SLEDAI-2k categories, n (%)	
No activity, n (%)	109 (40)
Mild, n (%)	107 (39)
Moderate, n (%)	41 (15)
High, n (%)	10 (4)
Very High, n (%)	4 (1)
Auto-antibody profile	
Anti-DNA positive, n (%)	151 (67)
Anti-ENA positive, n (%)	164 (69)
Anti-SSA, n (%)	55 (35)
Anti-SSB, n (%)	36 (21)
Anti-RNP, n (%)	64 (28)
Anti-Sm, n (%)	24 (10)
Anti-ribosome	13 (9)
Anti-nucleosome	32 (22)
Anti-histone	22 (15)
Antiphospholipid syndrome, n (%)	43 (16)
Antiphospholipid autoantibodies, n (%)	61 (32)
Lupus anticoagulant, n (%)	51 (28)
ACA IgM, n (%)	22 (11)
ACA IgG, n (%)	39 (20)
Anti beta2 glycoprotein IgM, n (%)	19 (10)
Anti beta2 glycoprotein IgG, n (%)	28 (15)
Current prednisone, n (%)	140 (50)
Prednisone, mg/day	5 (5–7.5)
Hydroxychloroquine, n (%)	194 (69)
Methotrexate, n (%)	31 (11)
Mycophenolate mofetil, n (%)	31 (11)
Azathioprine, n (%)	43 (15)
Rituximab, n (%)	8 (3)
Belimumab, n (%)	8 (3)

Data represent mean ± SD or median (interquartile range) when the data were not normally distributed. BMI: body mass index; C3 C4: complement; CRP: C reactive protein; LDL: low-density lipoprotein. DMARD: disease-modifying antirheumatic drug; ACA: anticardiolipin. HDL: high-density lipoprotein; ANA: antinuclear antibodies; Anti-ENA: extractible nuclear antibodies. SLEDAI: Systemic Lupus Erythematosus Disease Activity Index. SLEDAI categories were defined as 0, no activity; 1–5 mild; 6–10 moderate; >10 high; >20 very high activity. SLICC: Systemic Lupus International Collaborating Clinics/American College of Rheumatology Damage Index. IL-6: interleukin-6.

**Table 2:** Relationship between demographics and disease characteristics with IL-6 serum levels.

	IL-6 pg/mL	
	Beta Coef. (95%), *p*	
Age, years	0.07 (0.01–0.1)	0.014
Female	−2 (−5–(−0.03))	0.024
Body mass index, kg/m^2^	0.07 (−0.04–0.2)	0.20
Abdominal circumference, cm	0.04 (−0.005–0.08)	0.078
Hip circumference, cm	0.02 (−0.03–0.08)	0.36
Waist-to-hip ratio	9 (0.04–17)	0.041
Systolic pressure, mmHg	0.03 (−0.004–0.06)	0.084
Diastolic pressure, mmHg	0.04 (−0.02–0.09)	0.19
Cardiovascular co-morbidity		
Smoking	0.5 (−0.9–2)	0.47
Diabetes	0.9 (−2–3)	0.47
Hypertension	0.8 (−0.4–2)	0.19
Obesity	0.1 (−1–1)	0.86
Statins	0.8 (−0.6–2)	0.26
Aspirin	0.6 (−0.7–2)	0.37
SLE-related data		
Disease duration, years	0.06 (−0.004–0.1)	0.065
CRP, mg/dl	**0.2 (0.2–0.3)**	**<0.001**
SLICC-DI	0.2 (−0.2–0.5)	0.35
SLICC-DI ≥ 1, n (%)	1 (−0.3–2)	0.12
Katz Index	−0.2 (−0.5–0.2)	0.33
Katz ≥ 3	−0.6 (−2–0.6)	0.34
SLEDAI-2K	−0.01 (−0.2–0.1)	0.87
SLEDAI-2k categories		
No activity	-	-
Mild	−0.2 (−2–1)	0.83
Moderate to very high	−0.5 (−2–1)	0.60
Auto-antibody profile		
Anti-DNA positive	0.2 (−1–2)	0.75
Anti-ENA positive	0.3 (−1–2)	0.73
Anti-SSA	1 (−0.8–3)	0.23
Anti-SSB	0.6 (−3–5)	0.78
Anti-RNP	−0.6 (−2–0.9)	0.43
Anti-Sm	1 (−1–3)	0.42
Anti-ribosome	−0.6 (−4–3)	0.73
Anti-nucleosome	0.7 (−2–3)	0.56
Anti-histone	−0.7 (−3–2)	0.62
Antiphospholipid syndrome	−1 (−3–0.5)	0.17
Antiphospholipid autoantibodies		
Lupus anticoagulant	**2 (0.3–3)**	**0.016**
ACA IgM	2 (−1–4)	0.23
ACA IgG	−1 (−3–0.6)	0.18
Anti beta2 glycoprotein IgM	0.5 (−2–3)	0.71
Anti beta2 glycoprotein IgG	0.5 (−2–3)	0.65
Current prednisone	0.6 (−0.6–2)	0.32
Prednisone, mg/day	−0.05 (−0.3–0.2)	0.74
Hydroxychloroquine	−0.4 (−2–0.9)	0.56
Methotrexate	1 (−0.8–3)	0.26
Mycophenolate mofetil	−0.5 (−3–1)	0.60
Azathioprine	−0.8 (−3–1)	0.37
Rituximab	−1 (−4–4)	0.94
Belimumab	−2 (−6–2)	0.35

In this analysis, IL-6 was considered the dependent variable. ANA: antinuclear antibodies; Anti-ENA: extractible nuclear antibodies, ACA: anticardiolipin. SLEDAI-2k: Systemic Lupus Erythematosus Disease Activity Index. SLEDAI-2k categories were defined as 0, no activity; 1–5 mild; 6–10 moderate; >10 high activity, >20 very high activity. SLICC-DI: Systemic Lupus International Collaborating Clinics/American College of Rheumatology Damage Index. Significant *p*-values are depicted in bold.

**Table 3:** Relationship between individual disease score items and serum IL-6 levels.

			IL-6, pg/mL	
	n	%	Beta Coef. (95%)	*p*
Katz index				
History of cerebritis (seizure or organic brain syndrome)	12	6	−0.2 (−2–1)	0.77
History of pulmonary disease	10	5	0.4 (−1–2)	0.64
Biopsy proven diffuse proliferative glomerulonephritis	23	12	−1 (−2–0.1)	0.080
Four to six ARA criteria for SLE satisfied to date	139	73	0.3 (−1–2)	0.76
Seven or more ARA criteria for SLE satisfied to date	23	12	−0.88 (−2–0.4)	0.18
History of proteinuria (two or more)	62	32	−0.1 (−2–1)	0.87
Lowest recorded hematocrit to date = 30–37%	88	46	−0.1 (−2–1)	0.87
Lowest recorded hematocrit to date < 30%	47	25	−0.7 (−2–0.2)	0.12
Highest recorded creatinine to date = 1.3–3	28	15	0.7 (−1–3)	0.52
Highest recorded creatinine to date > 3	3	2	−1 (−5–2)	0.46
SLEDAI				
Seizures	1	0	1 (−9–11)	0.84
Psychosis	1	0	8 (−2–17)	0.12
Organic brain syndrome	0	0	-	
Visual disturbance	1	0	0.05 (−10–10)	0.99
Cranial nerve disorder	1	0	−3 (−12–7)	0.58
Lupus headache	1	0	-	
ACVA	0	0	-	
Vasculitis	1	0	6 (−4–15)	0.24
Arthritis	9	3	−2 (−5–2)	0.37
Myositis	0	0	-	
Urinary cylinders	7	3	3 (−1–8)	0.13
Hematuria	16	6	2 (−1–5)	0.23
Proteinuria	5	2	−2 (−7–4)	0.54
Pyuria	11	4	**4 (0.8–8)**	**0.017**
Rash	21	8	0.9 (−1–3)	0.46
Alopecia	11	4	−2 (−6–1)	0.18
Mucosal ulcers	14	5	−1 (−4–2)	0.37
Pleurisy	3	1	−2 (−11–8)	0.74
Pericarditis	1	0	−1 (−11–9)	0.84
Low complement	76	28	−0.6 (−2–0.8)	0.38
Elevated antiDNA	85	31	−0.6 (−2–0.7)	0.36
Fever	2	1	−0.2 (−7–7)	0.96
Thrombopenia	10	4	−1 (−4–2)	0.52
Leukopenia	19	7	−0.7 (−3–2)	0.58
SLICC domains				
Ocular	63	22	0.6 (−0.8–2)	0.40
Neuropsychiatric	40	14	−0.4 (−2–1)	0.69
Renal	28	10	−1 (−3–1)	0.30
Pulmonary	19	7	0.2 (−2–3)	0.86
Cardiovascular	23	8	1 (−1–3)	0.36
Peripheral vascular	34	12	−0.04 (−2–2)	0.97
Gastrointestinal	28	10	0.2 (−2–2)	0.85
Musculoskeletal	89	31	0.2 (−1–2)	0.81
Skin	39	14	1 (−0.3–3)	0.095
Premature gonadal failure	19	7	−0.2 (−3–2)	0.89
Diabetes (regardless of treatment)	18	6	0.9 (−1–3)	0.45
Malignancy (excluded dysplasia)	11	4	−0.5 (−3–2)	0.73

In this analysis, IL-6 was considered the dependent variable. Significant *p*-values are depicted in bold. History of pulmonary disease refers to the presence of lupus pneumonitis, pulmonary hemorrhage, or pulmonary hypertension. ARA: American Rheumatism Association; ACVA: acute cerebrovascular accident. SLEDAI: Systemic Lupus Erythematosus Disease Activity Index; SLE: systemic lupus erythematosus. SLICC: Systemic Lupus International Collaborating Clinics/American College of Rheumatology Damage Index. The presence of a SLICC domain involvement was shown if points in the domain were ≥1. See Table A1.

**Table 4:** Association between factors related to the cardiovascular system and IL-6 in patients with SLE.

		IL-6 pg/mL			
		Beta Coef. (95%), *p*			
		Univariate		Multivariate	
Lipid profile					
Cholesterol, mg/dL	198 ± 36	**−0.02 (−0.04–(−0.003))**	**0.024**	**−0.02 (−0.04–(−0.002))**	**0.026**
Triglycerides, mg/dL	130 ± 78	0.007 (−0.002–0.02)	0.12	0.005 (−0.004–0.01)	0.32
LDL cholesterol, mg/dL	111 ± 29	−0.02 (−0.04–0.0008)	0.059	−0.02 (−0.04–0.003)	0.097
HDL cholesterol, mg/dL	61 ± 19	**−0.05 (−0.08–(−0.01))**	**0.008**	**−0.04 (−0.08–(−0.01))**	**0.011**
LDL:HDL cholesterol ratio	1.96 ± 0.75	0.4 (−0.5–1)	0.40		
Non-HDL cholesterol, mg/dL	137 ± 33	−0.01 (−0.03–0.009)	0.32		
Lipoprotein (a), mg/dL	39 (12–108)	0.005 (−0.002–0.01)	0.16	0.004 (−0.003–0.01)	0.25
Apolipoprotein A1, mg/dL	173 ± 35	**−0.02 (−0.04–(−0.0008))**	**0.040**	**−0.02 (−0.04–(−0.001))**	**0.035**
Apolipoprotein B, mg/dL	95 ± 23	−0.001 (−0.03–0.03)	0.92		
ApoB:Apo A ratio	0.57 ± 0.17	2 (−1–6)	0.19	3 (−1–6)	0.16
Atherogenic index	3.5 ± 1.1	0.4 (−0.1–1)	0.14	0.4 (−0.2–1)	0.21
Insulin resistance indices *					
Glucose, mg/dL	91 ± 9	0.02 (−0.05–0.08)	0.65		
Insulin, µU/mL	6.6 (4.4–9.8)	0.07 (−0.006–0.1)	0.073	0.05 (−0.02–0.1)	0.16
C-peptide, ng/mL	2.2 (1.5–3.3)	0.1 (−0.08–0.3)	0.22		
HOMA2-IR	0.86 (0.59–1.127)	0.5 (−0.04–1)	0.066	0.4 (−0.1–1)	0.14
HOMA2-S%	116 (79–172)	−0.005 (−0.01–0.0008)	0.091	−0.004 (−0.01–0.002)	0.16
HOMA2-B%-C-peptide	156 ± 89	0.006 (−0.0008–0.01)	0.085	0.004 (−0.003–0.01)	0.28
Carotid ultrasound					
cIMT, mm	0.628 ± 0.109	**6 (0.4–11)**	**0.035**	4 (−3–10)	0.26
Carotid plaque	99 (36)	**1 (0.05–3)**	**0.041**	1 (−0.4–2)	0.15
SCORE2 calculator					
SCORE 2	2.1 (0.9–3.9)	**0.2 (0.02–0.4)**	**0.028**		
SCORE2 categories, n (%)					
Low to moderate	224 (79)	ref.			
High	50 (18)	1 (−0.5–3)	0.17		
Very high	18 (4)	−0.7 (−4–3)	0.68		

In this analysis, IL-6 was considered the dependent variable. IL-6: interleukin-6. Significant *p*-values are depicted in bold. LDL: low-density lipoprotein, HDL: high-density lipoprotein, cIMT: carotid intima thickness. HOMA: homeostatic model assessment, SCORE: Systematic Coronary Risk Assessment. * In this analysis, the relationship between insulin resistance indices and IL-6 was only performed in non-diabetes patients and glucose < 110 mg/dL (n = 221). Multivariate analysis was adjusted for age, gender, abdominal circumference, and hypertension. The relationship between SCORE2 and IL-6 was not adjusted for covariables because this score was calculated using several composite variables.

**Table 5:** Multivariate analysis of the relationship between the pathways and components of the complement system and IL-6.

		IL-6, pg/mL			
		Beta Coef. (95%CI), p			
		Univariate		Multivariate	
Classical pathway					
Functional assay, %	91 ± 38	0.01 (−0.006–0.03)	0.21		
C1q, mg/dL	34 ± 11	−0.03 (−0.09–0.03)	0.32		
Lectin pathway					
Functional assay, %	10 (1–41)	−0.0005 (−0.02–0.01)	0.95		
Common elements of the classic and lectin pathways					
C2, mg/dL	2.5 ± 1.2	−0.01 (−0.5–0.5)	0.96		
C4, mg/dL	21 ± 12	0.03 (−0.02–0.08)	0.27		
C1 inhibitor, mg/dL	32 ± 9	**0.07 (0.003–0.1)**	**0.040**	**0.07 (−0.0004–0.1)**	**0.049**
Alternative pathway					
Functional assay, %	41 (12–79)	0.01 (−0.001–0.03)	0.071	**0.02 (0.0002–0.03)**	**0.047**
Factor D, ng/mL	2593 ± 1836	0.0002 (−0.0002–0.0006)	0.41		
Common elements of the three pathways					
C3, mg/dL	130 ± 40	0.01 (−0.005–0.03)	0.20		
C3a, mg/dL	39 ± 10	**0.1 (0.05–0.2)**	**<0.001**	**0.1 (0.05–0.2)**	**<0.001**
Factor H, ng/mL × 10^−3^	389 (281–564)	−0.0003 (−0.001–0.0005)	0.46		

Complement routes and elements are considered the independent variable. Significant *p*-values are depicted in bold. The multivariate analysis was adjusted for age, gender, abdominal circumference, and hypertension.

## Data Availability

The data sets used and/or analyzed in the present study are available from the corresponding author upon request.

## Appendix A

**Table A1:** Relationship between SLICC score items and, IL-6.

			IL-6, pg/mL	
	n	%	Beta Coef. (95%)	*p*
Ocular				
Any cataract ever	29	11	**2 (0.02–4)**	**0.048**
Retinal change or optic atrophy	33	12	−0.2 (−2–2)	0.84
Points ≥ 1 in the domain	63	22	0.6 (−0.8–2)	0.40
Neuropsychiatric				
Cognitive impairment	7	3	−0.6 (−5–3)	0.78
Seizures requiring therapy for 6 months	15	5	−1 (−4–2)	0.41
Cerebrovascular accident ever	13	5	−0.8 (−3–2)	0.57
Cranial or peripheral neuropathy	5	2	4 (−1–9)	0.12
Transverse myelitis	1	0	−1 (−11–9)	0.82
Points ≥ 1 in the domain	40	14	−0.4 (−2–1)	0.69
Renal				
Estimated or measured glomerular filtration rate < 50%	13	5	−0.7 (−4–3)	0.68
Proteinuria 3.5 gm/24 h	7	3	−2 (−7–3)	0.34
End-stage renal disease	4	1	−0.4 (−2–1)	0.67
Points ≥ 1 in the domain	28	10	−1 (−3–1)	0.30
Pulmonary				
Pulmonary hypertension	4	1	3 (−1–8)	0.21
Pulmonary fibrosis	4	1	−1 (−6–4)	0.70
Shrinking lung	2	1	−3 (−12–7)	0.61
Pleural fibrosis	1	0	**10 (0.9–20)**	**0.033**
Pulmonary infarction	1	0	−3 (−12–7)	0.57
Points ≥ 1 in the domain	19	7	0.2 (−2–3)	0.86
Cardiovascular				
Angina or coronary artery bypass	4	1	1 (−4–6)	0.60
Myocardial infarction ever	2	1	−0.2 (−7–7)	0.96
Cardiomyopathy	2	1	7 (−3–16)	0.17
Valvular disease	9	3	2 (−0.8–6)	0.14
Pericarditis for 6 months, or pericardiectomy	2	1	0.06 (−10–10)	0.99
Points ≥ 1 in the domain	23	8	1 (−1–3)	0.36
Peripheral vascular				
Claudication for 6 months	3	1	−0.2 (−6–5)	0.95
Minor tissue loss (pulp space)	5	2	−0.5 (−6–5)	0.85
Significant tissue loss ever	0	0	-	
Venous thrombosis	14	5	0.8 (−2–4)	0.55
Points ≥ 1 in the domain	34	12	−0.04 (−2–2)	0.97
Gastrointestinal				
Infarction or resection of bowel	22	8	0.5 (−2–3)	0.66
Mesenteric insufficiency	1	0	−3 (−13–6)	0.49
Infarction or resection of bowel below duodenum, spleen, liver, or chronic peritonitis	1	0	−3 (−13–7)	0.55
Stricture or upper gastrointestinal tract surgery ever	0	0	-	
Pancreatic insufficiency	0	0	-	
Points ≥ 1 in the domain	28	10	0.2 (−2–2)	0.85
Musculoskeletal				
Muscle atrophy or weakness	3	1	−2 (−9–5)	0.60
Deforming or erosive arthritis	40	15	−0.5 (−2–1)	0.62
Osteoporosis with fracture or vertebral collapse	23	9	2 (−0.6–4)	0.14
Avascular necrosis	7	3	−0.5 (−5–4)	0.82
Osteomyelitis	1	0	0.06 (−10–10)	0.99
Tendon rupture	4	2	3 (−2–8)	0.19
Points ≥ 1 in the domain	89	31	0.2 (−1–2)	0.81
Skin				
Scarring chronic alopecia	16	6	0.7 (−2–3)	0.63
Extensive scarring or panniculum	10	4	−0.06 (−3–3)	0.97
Skin ulceration	4	1	**11 (6–16)**	**<0.001**
Points ≥ 1 in the domain	39	14	1 (−0.3–3)	0.095
Premature gonadal failure	19	7	−0.2 (−3–2)	0.89
Diabetes (regardless of treatment)	18	6	0.9 (−1–3)	0.45
Malignancy (exclude dysplasia)	11	4	−0.5 (−3–2)	0.73

SLICC items and domains represent the independent variable. SLICC: Systemic Lupus International Collaborating Clinics/American College of Rheumatology Damage Index. Significant *p*-values are depicted in bold.
